# New Insights Into Blue Light Phototherapy in Experimental *Trypanosoma cruzi* Infection

**DOI:** 10.3389/fcimb.2021.673070

**Published:** 2021-10-13

**Authors:** Natália Ivanova, Ana Luísa Junqueira Leite, Marcel Barbosa Vieira, Otto Henrique Cezar e Silva, Ludmilla Walter Reis Mota, Guilherme de Paula Costa, Cristiano Schetini de Azevedo, Sarah Alves Auharek, Romulo Dias Novaes, Kelerson Mauro de Castro Pinto, Rodrigo Fernando Bianchi, André Talvani

**Affiliations:** ^1^ Laboratório de Imunobiologia da Inflamação, Departamento de Ciências Biológicas, Instituto de Ciências Exatas e Biológicas (ICEB), Universidade Federal de Ouro Preto, Ouro Preto, Brazil; ^2^ Programa de Pós Graduação em Ecologia de Biomas Tropicais, Universidade Federal de Ouro Preto, Ouro Preto, Brazil; ^3^ Laboratório de Polímerose Propriedades Eletrônicas de Materiais, Departamento de Física, ICEB, Universidade Federal de Ouro Preto, Ouro Preto, Brazil; ^4^ Programa de Pós-graduação em Saúde e Nutrição, Universidade Federal de Ouro Preto, Ouro Preto, Brazil; ^5^ Faculdade de Medicina do Mucuri, Universidade Federal dos Vales do Jequitinhonha e Mucuri, Teofilo Otoni, Brazil; ^6^ Departamento de Biologia Estrutural, Universidade Federal de Alfenas, Alfenas, Brazil; ^7^ Escola de Educação Física, Universidade Federal de Ouro Preto, Ouro Preto, Brazil; ^8^ Programa de Pós-graduação em Infectologia e Medicina Tropical, Universidade Federal de Minas Gerais, Belo Horizonte, Brazil

**Keywords:** *Trypanosoma cruzi*, inflammation, blue light, phototherapy, cardiac disease

## Abstract

The search for an effective etiologic treatment to eliminate *Trypanosoma cruzi*, the causative agent of Chagas disease, has continued for decades and yielded controversial results. In the 1970s, nifurtimox and benznidazole were introduced for clinical assessment, but factors such as parasite resistance, high cellular toxicity, and efficacy in acute and chronic phases of the infection have been debated even today. This study proposes an innovative strategy to support the controlling of the *T. cruzi* using blue light phototherapy or blue light-emitting diode (LED) intervention. In *in vitro* assays, axenic cultures of Y and CL strains of *T. cruzi* were exposed to 460 nm and 40 µW/cm^2^ of blue light for 5 days (6 h/day), and parasite replication was evaluated daily. For *in vivo* experiments, C57BL6 mice were infected with the Y strain of *T. cruzi* and exposed to 460 nm and 7 µW/cm^2^ of blue light for 9 days (12 h/day). Parasite count in the blood and cardiac tissue was determined, and plasma interleukin (IL-6), tumoral necrosis factor (TNF), chemokine ligand 2 (CCL2), and IL-10 levels and the morphometry of the cardiac tissue were evaluated. Blue light induced a 50% reduction in *T. cruzi* (epimastigote forms) replication *in vitro* after 5 days of exposure. This blue light-mediated parasite control was also observed by the *T. cruzi* reduction in the blood (trypomastigote forms) and in the cardiac tissue (parasite DNA and amastigote nests) of infected mice. Phototherapy reduced plasma IL-6, TNF and IL-10, but not CCL2, levels in infected animals. This non-chemical therapy reduced the volume density of the heart stroma in the cardiac connective tissue but did not ameliorate the mouse myocarditis, maintaining a predominance of pericellular and perivascular mononuclear inflammatory infiltration with an increase in polymorphonuclear cells. Together, these data highlight, for the first time, the use of blue light therapy to control circulating and tissue forms of *T. cruzi*. Further investigation would demonstrate the application of this promising and potential complementary strategy for the treatment of Chagas disease.

## Introduction

Chagas disease is the main cause of myocarditis in Latin America and worldwide (Rassi et al., 2010). Chemotherapy strategies using benznidazole or nifurtimox have been proposed to eliminate its causative agent, the protozoan *Trypanosoma cruzi*, through the production of cytotoxic free radical intermediates and metabolites generated by nitroreductases (Urbina, 2009; Kratz et al., 2018). However, even after a century of Chagas disease discovery, approximately 7.5 million individuals are still infected with this protozoan of which less than 18,000 patients are under chemotherapy (Echeverría et al., 2020). *T. cruzi*-induced cardiomyopathy is the cause of 12,000 deaths per year. Further, the high cytotoxicity of both medicines, the close dependency of their effects on the genetic background of the parasite, and the real benefits of the precocious (acute phase: trypanocide effects) or the late (chronic phase: cardiac protection or stabilization) treatment have still created fragile barriers concerning the clinical management of Chagas disease (Morillo et al., 2015; Zingales, 2018).

New chemotherapy targets have focused on azole inhibitors of sterol 14alpha-demethylase (CYP51) or other natural compounds (Molina et al., 2014; Martínez-Peinado et al., 2020; Menezes et al., 2020), but all experimental and/or clinical trial findings have not been successful.

Therefore, a new physical strategy using blue light-emitting diode (LED) phototherapy is proposed to support chemotherapy against *T. cruzi*. Blue light is part of the natural light received from the sun. In addition, humans are also indirectly exposed to blue light from electronic devices such as computers and phone screens. For decades, blue light has successfully improved new-born jaundice therapies in medical centers (Porto and Hsia, 1969; Ferreira et al., 2009). Beyond this application, phototherapy is also capable of inactivating microorganisms such as bacteria and fungi (Trzaska et al., 2017; Lewis et al., 2018). The proposed action of this light on susceptible organisms is based on the photoexcitation of endogenous porphyrins that increases the level of reactive oxygen species (Wu et al., 2018) and/or causes damage to proteins and lipids from the microorganism’s membrane, thereby affecting intracellular transport (Alves et al., 2013). An additional effect of blue light is photo-immunomodulation because light, in general, affects the release of hormones and cytokines (Walton et al., 2011; Yuan et al., 2016).

In this study, we investigated whether blue light could interfere in the *T. cruzi* biological activities at a wavelength of approximately 460 nm. We first show the ability of blue light to inhibit *T. cruzi* replication *in vitro*, and then demonstrate its capacity to cross the skin of infected mice and inhibit the replication of blood-borne and tissue-borne parasites in addition to exhibiting beneficial cardiac immune-modulatory effects.

## Material and Methods

### Ethical Approval

All the methodologies performed in this study were in accordance with the standards of the National Council for Control of Animal Experimentation (CONCEA) and previously approved by the Animal Research Ethics Committee (CEUA) of the Federal University of Ouro Preto (UFOP), Ouro Preto, Minas Gerais, Brazil, under the protocol number 089/2018.

### *T. cruzi* Infection

For these experiments, we used the Y and CL strains of *T. cruzi*, classified as *T. cruzi* II and *T. cruzi* VI, respectively (Zingales et al., 2009). These strains were maintained by successive passages in Swiss mice at the Center of Animal Science, UFOP.

### Culture Assays

Pure cell cultures of *T. cruzi* (Y and CL strains) were maintained at 25°C in liver infusion tryptose (LIT) broth medium (Becton-Dickinson, NJ, EUA) supplemented with 10% (v/v) inactivated fetal bovine serum (Sigma-Aldrich, San Luis, MS, EUA). These cultures were exposed to blue LED light (460 nm and 40 µW/cm^2^) for 5 days, 6 h per day, at 24°C. The parasites were counted in 10 µL volume every day for quantification and analysis of their survival using the Neubauer chamber. All the experiments were repeated twice.

### Animals and *T. cruzi* Infection

**Figure 1:**
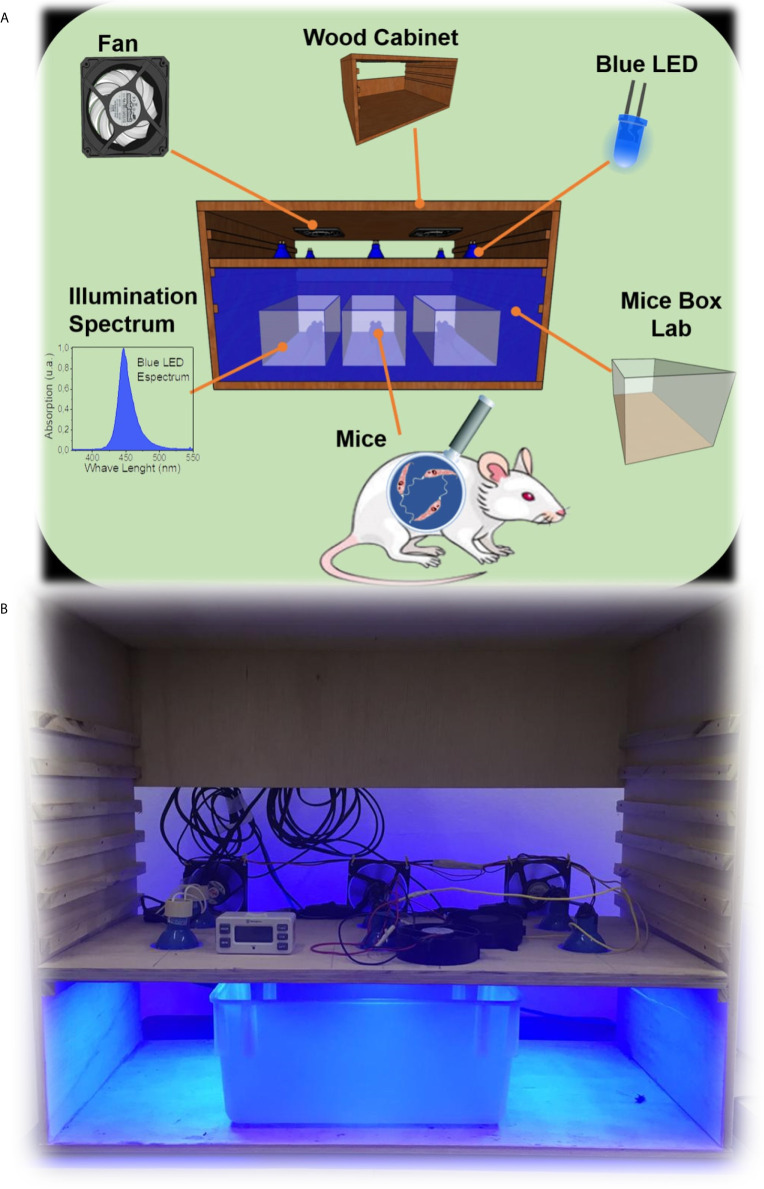
**(A)** Schematic illustration of homebuilt system for the blue-light phototherapy. Mice were infected with Y strain of *T. cruzi* and exposed to 460 nm and 7 µW/cm^2^ of blue light for 9 days (12h/day). A fan was used to maintain a constant temperature in the mice box. **(B)** A photo of this robust system is inserted in the figure.

C57BL/6 male mice aged 20-22 weeks weighing approximately 30 g were used in this study. Animals (n = 10) were grouped as (i) non-infected + conventional light, (ii) non-infected + blue light, (iii) *T. cruzi*-infected + conventional light, and (iv) *T. cruzi*-infected + blue light. Animals were infected by an intraperitoneal injection of the Y strain of the parasite (100 trypomastigotes/animal). During this phase of the experiment, animals exposed to the phototherapy received blue LED light (460 nm and 7 µW/cm^2^) for 9 days, 12 h per day (from 07:00h to 19:00h), at 26°C using a homebuilt system (**Figure 1**). Blood parasites were daily evaluated in infected mice according to Brener’s method (Brener, 1962). On day 9 of infection, the animals were euthanized, their blood and hearts were collected for the immune assay and histopathology analysis, respectively. The mice were housed and maintained at the Center of Animal Science at UFOP.

### *T. cruzi* DNA Extraction in the Cardiac Tissue

The *T. cruzi*-genomic DNA was extracted from 10 mg of cardiac tissue at 9^th^ day after infection, using the Wizard^®^ SV Genomic DNA Purification System kit (Promega, Wisconsin, EUA) according to the manufacturer’s instructions. Real-time polymerase chain reaction (PCR) was performed to quantify the tissue parasitism as previously described (Cummings and Tarleton, 2003), using the *T. cruzi* (sense: *AAATAATGTACGGG(T/G)GAGATGCATGA* and antisense: *GGGTTCGATTGGGGTTGGTGT*). Each DNA sample was quantified in triplicate.

### Enzyme-Linked Immunosorbent Assay

Immunoenzymatic assays were used to evaluate the plasma concentrations of interleukin (IL)-6, tumor necrosis factor (TNF), chemokine ligand 2 (CCL2), and IL-10. Equal volumes of plasma samples and 1.2% trifluoroacetic acid/1.35 M sodium chloride (NaCl) (Sigma-Aldrich, San Luis, MS, EUA) were mixed and left at 24°C for 10 min. Samples were then centrifuged for 10 min at 1500 ×*g* and 4°C, and the supernatant obtained was adjusted for salt content [0.14 M NaCl (0.01 M) and sodium phosphate]. The pH was adjusted to 7.4, and the samples were added to wells of a 96-well plate previously coated with monoclonal antibodies specific for TNF, IL-6, CCL2, and IL-10 (PeproTech, Cranbury, NJ, USA) according to the manufacturer’s protocols. All samples were simultaneously measured in duplicates. Samples were read on a spectrophotometer (Emax Molecular Devices, Sunnyvale, CA, USA) at 405 and 630 nm wavelengths.

### Heart Processing and Histopathology

Heart samples of all animals were fixed in formalin for 24 h, dehydrated in ethanol, embedded in glycol methacrylate resin, and cut into 3 μm thick sections using glass knives (RM2125RTS1, Leica Biosystems, Wetzlar, Germany). Two histological slides with four heart sections collected in a semi-series were obtained using one out of every 50 sections to avoid evaluating the same histological area. The sections were stained with hematoxylin and eosin (H&E) at 60°C (40 min for conventional histopathology on the first slide). The distribution and organization of the heart parenchyma and connective stroma, tissue necrosis, and morphology and distribution of cardiomyocytes, blood vessels, and inflammatory foci were evaluated according to Novaes et al. (2013). The heart microstructure was analyzed by bright-field microscopy using a 40× objective lens (400× magnification; Axioscope A1, Carl Zeiss, Germany).

### Heart Stereology and Histomorphometry: Myocardial Compartments and Tissue Cellularity

Cardiac microstructural reorganization was analyzed following stereological principles according to Novaes et al. (2013). The volume density (Vv, %) of the heart stroma [connective tissue (CT)], parenchyma [cardiomyocytes (CMY)], and blood vessel (BV) was estimated as Vv = ΣPP/PT, where ΣPP is the number of test points on the structure of interest and PT is the total number of test points. A quadratic test system with 100 test points distributed in a standard test area of 25 × 103 μm^2^ was applied. Volume density was estimated from 20 randomly sampled histological fields of heart sections from each animal using a ×40 objective lens (400× magnification; Axioscope A1, Carl Zeiss, Germany). A total tissue area of 25.0 × 105 μm^2^ was analyzed for each group. In addition, heart sections were stained with hematoxylin at 60°C to evaluate myocardial cellularity and cardiomyocyte parasitism. The number density (QA, n/mm^2^) of mononuclear (MN) and polymorphonuclear (PMN) interstitial cells per myocardial area was estimated as QAMN/PMN = ΣQMN/PMN/AT, where ΣQMN/PMN is the number of MN or PMN cells in the microscopic focal plane and AT is the dimension (mm^2^) of the two-dimensional test area (AT = 0.009 mm^2^). QA was estimated from 20 randomly sampled histological fields from each animal using a ×100 objective lens (1000× magnification; Axioscope A1, Carl Zeiss, Germany), totaling 85,600 μm^2^ for each group of animals. The number of amastigote forms of *T. cruzi* per area was estimated using computational planimetry. Briefly, the number of amastigotes was counted and normalized by the nest area, which was determined using a contour function applied to the image analysis software. All morphological parameters were quantified using the image analysis software Image-Pro Plus 4.5 (Media Cybernetics, Rockville, Maryland, USA).

### Statistical Analysis

Data are expressed as mean ± standard error of means (SEM). Multiple groups were compared using one-way analysis of variance (ANOVA) followed by the Tukey-Kramer post-test. The survival rate was compared using the log-rank test (Mentel-Cox), and Student’s *t*-test was used to compare differences between two experimental groups. All analyses were performed using the Prism 8 software (GraphPad Software). Groups (6-10 surviving animals) were considered statistically different at *p* < 0.05.

## Results

### Effect of Blue Light on *T. cruzi* Axenic Culture

**Figure 2:**
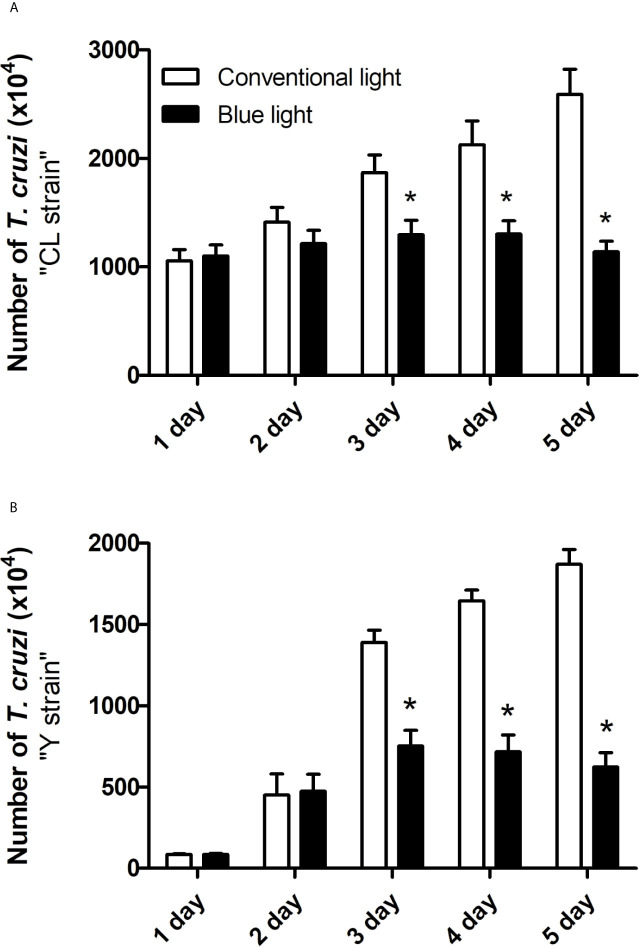
Growth inhibition of the CL **(A)** *and Y* **(B)** *strains of T. cruzi.* The parasites cultures were exposed to phototherapy with blue light for 5 days 6h per day, at room temperature. The growth inhibition was evaluated in 10 µL every day using the neubauer chamber and a light microscope. *p<0.05 means differences between control groups and blue light therapy.

To determine the effect of blue light on the isolated forms of these trypanosomatids, epimastigote forms of CL (**Figure 2A**) and Y (**Figure 2B**) strains of *T. cruzi* were incubated for 5 days (6 h/day) under blue light or conventional environmental light. Differences in the replication of cultured parasites were observed in both strains from day 3 until day 5 of incubation.

### Effect of Blue Light on *In Vivo T. cruzi* Infection

**Figure 3:**
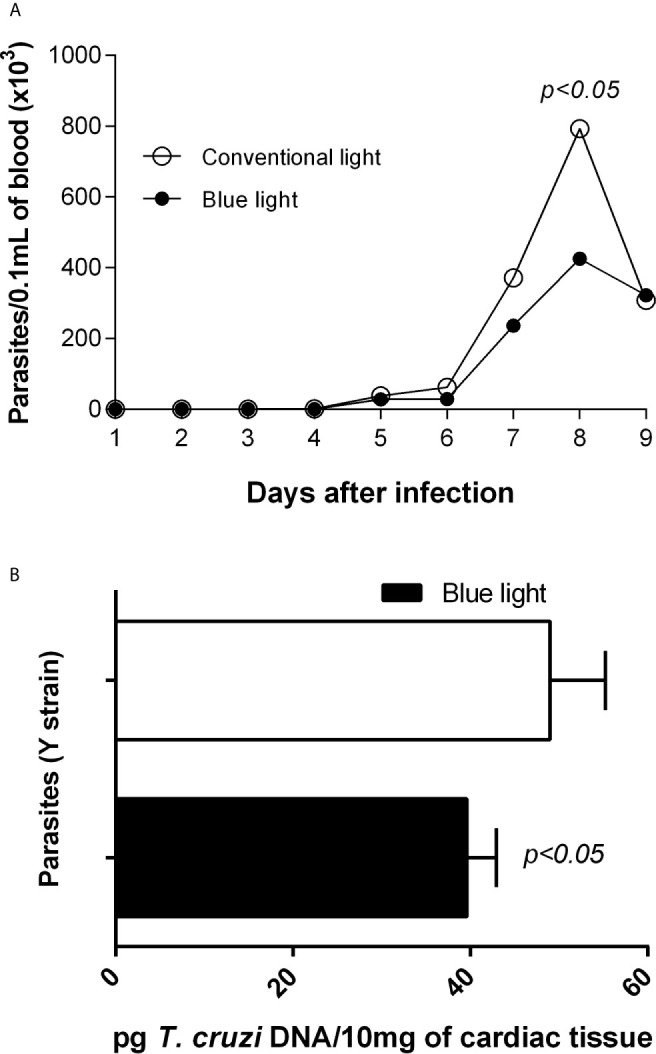
Parasite quantification and animals’ survival. **(A)** - curve of parasitemia; **(B)** *T. cruzi* genomic DNA quantification. C57BL/6 male mice of age 20-22 weeks and mass around 30 g were infected with Y strain of the *T. cruzi*. Then groups with “blue light” were exposed to blue light (with the peak 460 nm and 7 µW/cm2) for 9 days, 12 h per day (from 07:00h to 19:00h). For creating parasitemia curve, blood samples were collected every day and parasites number presented as average. For tissue parasitism, *T. cruzi* DNA was quantified in 10mg of cardiac tissue by PCR assay. p<0.05 means differences between groups under conventional and blue light therapy.

To verify the reproducibility of the effects of blue light on parasites in cultures, we infected mice with the Y strain of *T. cruzi* and exposed them to blue light at 12 h/day (**Figure 1**) for 9 days. There was an evident decrease in the number of blood-borne parasites from day 8 (**Figure 3A**), the peak of parasitemia, after infection. In addition, the *T. cruzi*-genomic DNA was quantified in the cardiac tissue and blue light was also capable to reduce the parasitism (**Figure 3B**) when compared with animals under conventional light.

### Blue Light-Mediated Changes in The Plasma Levels of Inflammatory Mediators

**Figure 4:**
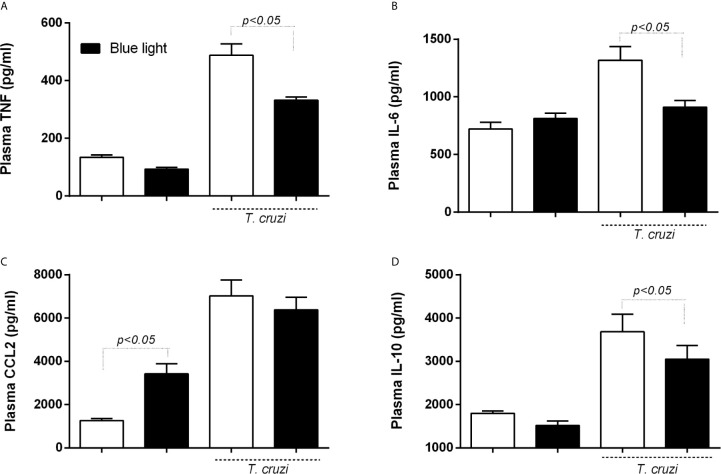
Level of plasma cytokines in C57BL/6 mice under blue light phototherapy. *T. cruzi*-infected animals were exposed to conventional and blue light for 9 days, 12 h per day. The plasma was collected to evaluate the cytokines TNF **(A)**, IL-6 **(B)**, CCL2 **(C)** and IL-10 **(D)** production. p<0.05 means differences between infected groups under blue light and conventional light.

Assuming the immune response as the key mechanism underlying the control of *T. cruzi* infection, we evaluated the plasma levels of inflammatory mediators TNF, IL-6, CCL2, and IL-10 (**Figure 4**) in uninfected and infected animals under blue light therapy. We observed an increase in the plasma level of TNF (**Figure 4A**) and IL-6 (**Figure 4B**) after *T. cruzi* infection. Blue light therapy, however, decreased the levels of both inflammatory cytokines.

Surprisingly, CCL2 level (**Figure 4C**) increased in the plasma of uninfected mice under blue light therapy. After *T. cruzi* infection, we observed higher production of this chemokine. Phototherapy failed to change CCL2 levels in the plasma of euthanized animals after 9 days of infection. Finally, *T. cruzi*-infected animals showed an increase in the plasma level of IL-10 (**Figure 4D**), which was partially reduced after blue light therapy.

### Cardiac Microstructural Reorganization After Blue Light Phototherapy

**Figure 5:**
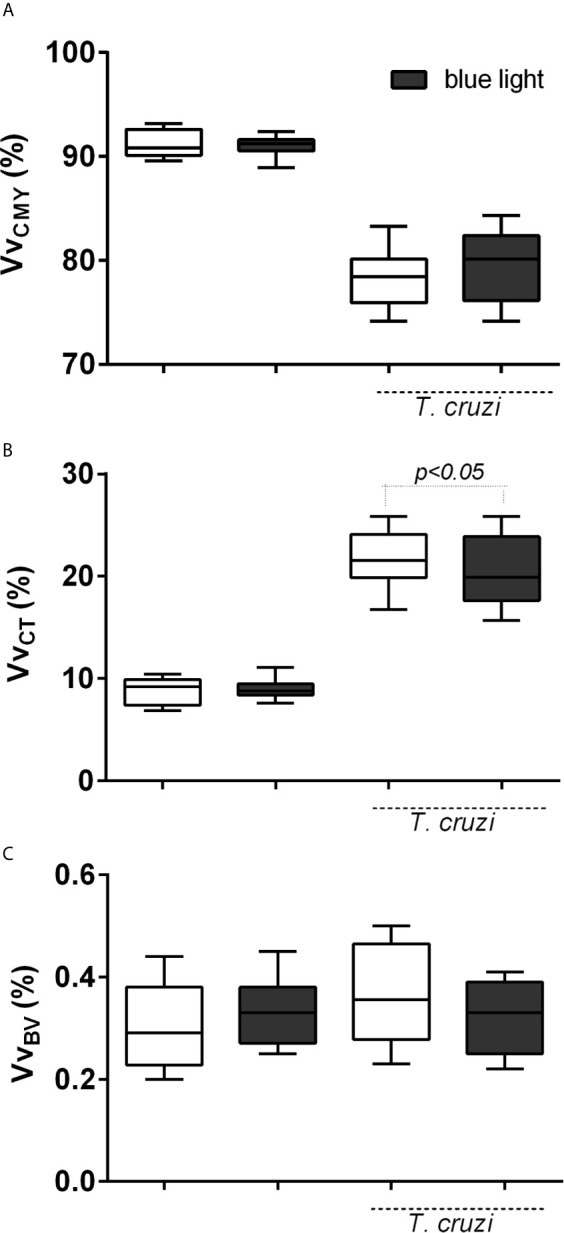
Cardiac microstructural reorganization according to stereological principles. **(A)** Volume density (Vv, %) of the heart stroma [connective tissue (CT)], **(B)** parenchyma [cardiomyocytes [CMY)], and **(C)** blood vessels (BV). p<0.05 means differences between groups under conventional and blue light therapy.

To verify whether blue light interfered with the cardiac microstructural reorganization, we evaluated stereological parameters. In the presence of *T. cruzi* infection, the volume density of cardiomyocyte parenchyma decreased (**Figure 5A**) and phototherapy had no effect on this parameter in uninfected or infected animals (**Figure 5A**). We investigated the volume density of the connective tissue in the cardiac stroma and found it to be significantly increased after parasite infection (**Figure 5B**). Blue light therapy could reduce this parameter in infected animals, suggestive of the protective effect on the organization of the cardiac stroma. Finally, we investigated the blood vessel volume density (**Figure 5C**) and observed no differences between uninfected and *T. cruzi-*infected animals subjected to blue light therapy.

### Effects of Blue Light Therapy on the Tissue-Borne Parasite and Cardiac Inflammatory Phenotype

**Figure 6:**
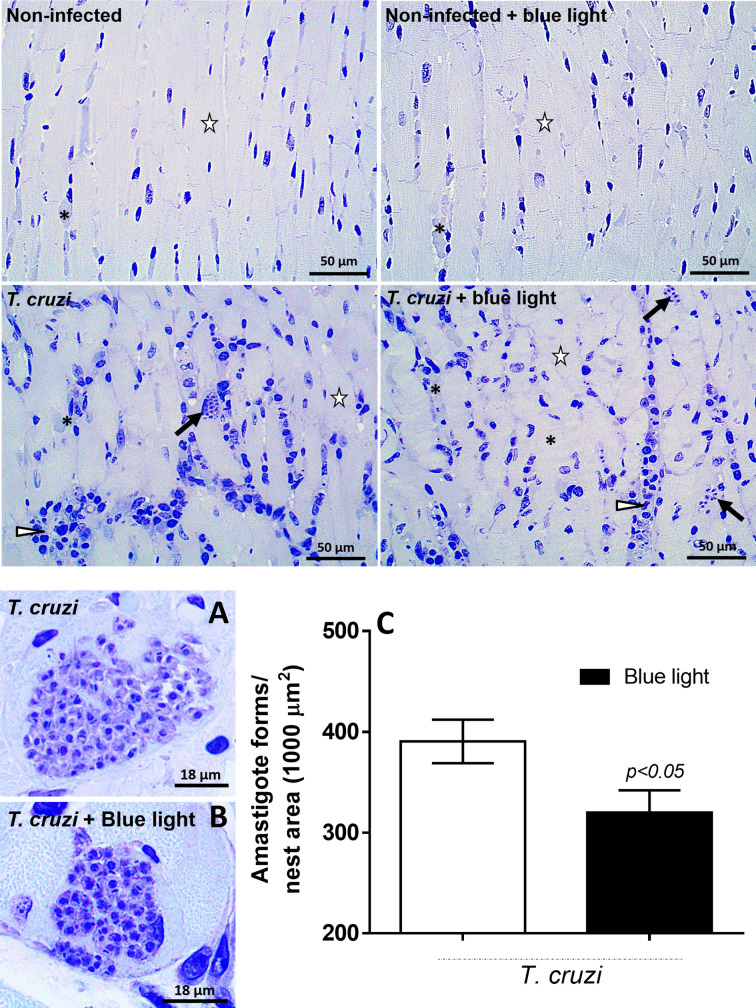
Quantification of amastigote nests in cardiac tissue. The amastigote nests of the *T. cruzi* were evidenced in the upper images with reduction of visual parasites in the infected group under blue light therapy. **(A)** An amastigote nest from animals under conventional light therapy, **(B)** an amastigote nest from animals under blue light therapy and **(C)** quantification of the area of the amastigote nests. p<0.05 means differences between infected groups under conventional and blue light therapy. Arrow – the *T. cruzi* amastigote nest, arrowhead – the inflammatory foci, asterisk – the blood vessel.

As blue light could control the parasite replication in cultures and circulating blood, we evaluated whether this effect was also evidenced on amastigotes, the evolutive form of the parasite in tissues that is prevalent in the chronic stage of the infection. **Figure 6** (upper side) shows a panel of cardiac tissue photomicrographs from uninfected and *T. cruzi*-infected animals under blue light therapy. We observed inflammatory infiltration and amastigote nests in the tissues infected with *T. cruzi*. We also noted the difference in the area of cardiac amastigote nest in an animal infected with *T. cruzi* (**Figure 6A**) and another infected animal under blue light phototherapy (**Figure 6B**). To confirm this observation, the area of amastigote nests in the cardiac tissue from both groups of infected animals (with and without blue light therapy) was plotted (**Figure 6C**).

**Figure 7:**
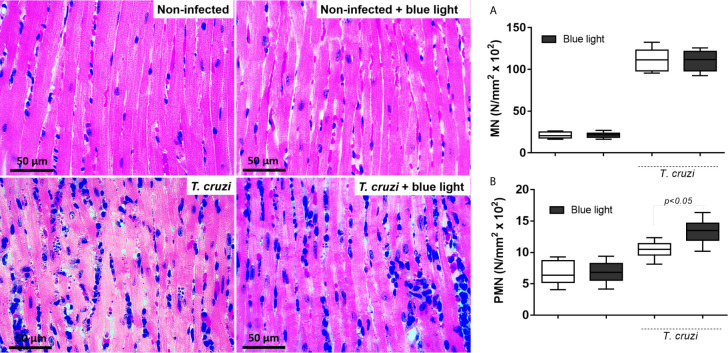
Photomicrography of cardiac tissue and analysis of the inflammatory infiltration. Cardiac tissue from non-infected and *T. cruzi*-infected animals were analyzed under conventional light and blue light phototherapy. Inflammatory cells and loss of tissue integrity are evidenced in those images related to parasite infection. Cardiac tissue from animals under conventional and blue light phototherapy was analyzed and mononuclear cells **(A)** polymorphonuclear cells **(B)** quantified. p<0.05 means differences between infected groups under blue light and conventional light.

Considering the smaller area of amastigote nests in infected animals under blue light therapy, we investigated the phenotype profile of inflammatory infiltrates in the cardiac tissue. As shown in **Figure 7** (on the left), the inflammatory infiltration was high in the animals infected with *T. cruzi*. However, quantification of inflammatory cells based to their phenotypes revealed no difference in MN cell population (**Figure 7A**) but a clear increase in the number of PMN cells (**Figure 7B**) in the infected animals under blue light therapy.

## Discussion

To our knowledge, this is the first study to demonstrate that blue light can interfere in biological parameters and in the controlling of the CL and Y strains of *T. cruzi* in cultures and blood of infected mice. Daily exposure to blue light modulated the murine immune response and reduced the quantification of parasites in the blood and in the cardiac tissue. This study proposes an alternative conception of the understanding of Chagas disease therapy with an insight into how phototherapy could be applied in support of benznidazole, nifurtimox, or any other compound proposed to treat Chagas disease.

The exposure of bacteria, mycoplasma, and viruses to a range of distinct light wavelengths can effectively inactivate them, thus serving as an important support for the medical management of infectious diseases. The investigation of the mechanisms underlying the antimicrobial effects of blue light has pointed out some evidence as follows: (i) an intracellular photosensitizing chromophore that can increase the level of reactive oxygen species and induce the death of the infective agent, depending on the optimal blue light therapeutic wavelength (Wu et al., 2018; Bumah et al., 2020); (ii) modulation in the structure of lipids and proteins that may affect the intracellular transport and organization of cell organelles (Alves et al., 2013; Wang et al., 2017); and (iii) modification in the humoral and cellular immune response of mammals (Yuan et al., 2016; Yu et al., 2019). Indeed, the antimicrobial effects of blue light therapy have differed among studies and the target microorganism species, possibly owing to environmental conditions, blue light wavelength spectrum, and time of light exposure. Based on the reported blue light effects on the elimination of resistant bacteria and/or fungi (Enwemeka, 2013; Rapacka-Zdonczyk et al., 2019), we investigated the action of blue light on the protozoan *T. cruzi*.

The chemotherapy proposed to treat Chagas disease has serious limitations such as (i) strong side-effects that interfere with treatment compliance of patients; (ii) resistance to some genetic populations of *T. cruzi*; (iii) distinct behavior of the evolutive forms of parasites in the blood and tissue and the consequent resistance to chemotherapy; and (iv) particularities related to the treatment in non-adult individuals and infected pregnant women (Carlier et al., 2011; Morillo et al., 2015; Jackson et al., 2020). Other limitations include failure of the translation of the chemical compound/drug tested in an experimental model to human subjects, owing to differences in pharmacological pathways (Ribeiro et al., 2020). For these reasons, safety alternatives that eliminate parasites and/or control *T. cruzi*-induced myocarditis are warranted, and blue light phototherapy is a potential strategy in this direction.

Given its proven safety and efficiency, blue light has already been included in the standard protocol for the treatment of human hyperbilirubinemia and as a therapy against diseases caused by infectious agents (Ammad et al., 2008; Pavie et al., 2019; Zhou et al., 2019). In the present study, *T. cruzi* appeared to be vulnerable to blue light therapy. This observation was based on the parasite (Y and CL strains) reduction in axenic cultures and compromised *T. cruzi* motility (data not shown). The anti-*T. cruzi* chemotherapy is challenged by mutations such as those in the genes encoding CYP51 or mitochondrial nitroreductase (*TcNTR-1*) in parasites, contributing to Chagas disease therapy failure (Campos et al., 2017). Assuming that this new physical intervention could minimize changes in the parasite genes, blue light therapy even with a partial action on parasites may potentialize the expected effects of benznidazole, nifurtimox, posaconazole, feninidazole, diamidines, naphthoquinones, xanthenodiones, and other anti-*T. cruzi* targets.

The direct effect of blue light on parasites in culture plates could predict their potential actions, as there were no physical, chemical, or biological barriers to this light. However, these effects were reproducible in infected mice and did not discriminate between the evolutive trypomastigote and amastigote forms. Of note, only a few studies have investigated the effect of photodynamic therapy based on the application of a photosensitive substance (e.g., methylene blue) on the target area, resulting in activation of oxidative reactions and the consequent death of pathogens (Gironés et al., 2006; Volpe et al., 2018; Barbosa et al., 2020). In the present study, we investigated the direct use of blue light at a single frequency and without any photosensitive substance against the parasite; blue light has an accumulative effect because changes were evident only after some days of phototherapy in both *in vitro* and *in vivo* assays.

Chronic parasite persistence in mammals is a consequence of the early development of intracellular amastigote forms of *T. cruzi* in the cardiac tissue, mechanical ruptures, and release of parasites (blood trypomastigote forms) and their residues, leading to the activation and recruitment of inflammatory cells into the heart. Inflammatory mediators clearly mediate part of the immune process, and elimination of the parasite or minimization of the host immune response could retard or partially prevent myocarditis. Another interesting finding was that phototherapy with blue light reduced the levels of inflammatory TNF, IL-6 and, consequently, the regulatory IL-10 in the plasma of *T. cruzi*-infected animals. However, at this time, the CCL2 was not affected by the blue light in those infected animals even assuming its relevance to the recruitment of mononuclear cells into the cardiac tissue in *T. cruzi* infection.

TNF, IL-6 and gamma-interferon (IFN-γ) can act in synergism to the optimal nitric oxide production to eliminate intracellular parasites in infected mammalian cells and activate other inflammatory signaling pathways promoting cardiac tissue damage in *T. cruzi* infected mice (Talvani et al., 2000). On the other hand, the regulatory cytokine IL-10 exerts control of the leukocyte activation/chemoattracting by inhibition of the NF-kB and ERK/MAPK, which minimize the cardiomyocytes damage induced by the parasite infection (Rada et al., 2020). By our own experience, IL-10 is shown overproduced in experimental *T. cruzi* infection following an increase of systemic and local inflammatory pattern in the infected animals. In this present study, the immunomodulatory effects of the blue light on the TNF and IL-6 and, on the reducing the amount of blood and tissue parasites could be, in part, related to the observed IL-10 reduction. Our group has investigated the use of different medicines in clinical practice (simvastatin, doxycycline, angiotensin-enzyme converter inhibitor, beta-blocker, and others) alone or in association with benznidazole to reduce the release of cytokines IFN-γ, TNF, IL-6, IL-10 and chemokines (CCL2, CCL3, CCL4, CCL5, CXCL9, CXCL11, CCL1, CCL17, CCL20, CCL24, and CCL26) and minimize inflammatory infiltration into the heart, which is important to ameliorate the consequences of *T. cruzi* antigens. However, these events trigger fibrosis formation and cardiac morphological and functional damage (Silva et al., 2012; de Paula Costa et al., 2016; Horta et al., 2017; Leite et al., 2017). These cytokines can shift the regulation patterns described during the acute and chronic courses of *T. cruzi* infection, affecting the parasite-host equilibrium and driving the pathogenesis process (Talvani and Teixeira, 2011). This new perspective of phototherapy was demonstrated to be effective in minimizing the parasitological and immunological targets, and could be further improved in the future.

In conclusion, our study demonstrates the susceptibility of *T. cruzi* to blue light therapy (460 nm and 40 µW/cm^2^), even more in axenic cultures than in experimentally infected mice. Blue light treatment reduced the quantification of distinct (Y and CL) strains of *T. cruzi* and reduced blood and intracellular forms of the parasite in mammalian hosts. These data support the importance of an alternative and/or complementary therapy against *T. cruzi* and instigate further investigations on the underlying mechanism and whether long-term phototherapy mitigates myocarditis diagnosed in experimental and human infections.

## Data Availability Statement

The raw data supporting the conclusions of this article will be made available by the authors, without undue reservation.

## Ethics Statement

The animal study was reviewed and approved by Animal Research Ethics Committee (CEUA) of the Federal University of Ouro Preto (UFOP), Ouro Preto, Minas Gerais, Brazil, under protocol number 089/2018.

## Author Contributions

AT, RFB, KMCP, CSA and NI designed the experiments and analyzed the data. NI, SAA, RDN, ALJL, MBV, LWRM, KMCP and GPC performed the experiments. AT, RB, CSA and NI wrote the paper with inputs from all co-authors. All authors contributed to the article and approved the submitted version.

## Funding

This research was supported by the UFOP, CNPq, Coordination of Improvement of Higher Education Personnel (CAPES), Minas Gerais Research Funding Foundation (FAPEMIG), and PEPROTECH US/Brazil.

## Conflict of Interest

The authors declare that the research was conducted in the absence of any commercial or financial relationships that could be construed as a potential conflict of interest.
